# Safety of intracameral injection of levofloxacin 0.5% eye drops single dose 0.6 ml preservative free on rabbit eye

**DOI:** 10.12688/f1000research.133293.2

**Published:** 2023-12-04

**Authors:** Lukman Edwar, Baltazar B. Bisara, Rianto Setiabudi, Eka Susanto, Gabriella H. Badruddin

**Affiliations:** 1 Ophtalmology Departement, Faculty of Medicine, Universitas Indonesia, Depok, West Java, 16424, Indonesia; 2 Pharmacology and Therapeutics Departement, Faculty of Medicine, Universitas Indonesia, Depok, West Java, 16424, Indonesia; 3 Anatomic Pathology Departement, Faculty of Medicine, Universitas Indonesia, Depok, West Java, 16424, Indonesia

**Keywords:** Levofloxacin 0.5% eye drops, Intracameral, Safety, Rabbit

## Abstract

**Background:**

This was an experimental, parallel, and randomized study to evaluate the safety of single intracameral injection of 0.6 ml 0.5% preservative-free levofloxacin eye drops on rabbit eye.

**Methods:**

In total, 24 eyes of 12 New Zealand white rabbits were divided into three groups. The first group (LFX) was treated with 0.1 ml intracameral injection of levofloxacin 0.5% eye drops of 0.6 ml preservative-free (n = 6), the second group (CRAV) was treated with 0.1 ml intracameral injection of levofloxacin 0.5% eye drops 5 ml commercially available eye drops preservative-free (n = 6), and the third group (BSS) were treated with 0.1 ml intracameral injection of *balanced salt solution* (n = 12). All groups received a single dose. The clinical evaluation was performed on the 1 ^st^, 3 ^rd^, 5 ^th^, and 7 ^th^ day after injection. Each eye was enucleated on the 7 ^th^ day and underwent a histopathology examination.

**Results:**

The clinical scores among the three groups did not show any significant difference on days 1 ^st^, 2 ^nd^, 3 ^rd^, and 7 ^th^ (p>0.05). The only ones noted in clinical scores were mild corneal opacity, mild cells, and flares in the anterior chamber. The histopathology score demonstrated no statistically significant difference between the three groups (p>0.05). Vacuolization of corneal endothelial cells was noted in all groups but was not statistically significant.

**Conclusions:**

A single intracameral injection of 0.6 ml 0.5% preservative-free levofloxacin eye drops was safe for rabbit eye, according to clinical and histopathology scores, similar to levofloxacin 0.5% eye drops in 5 ml bottle preservative free.

## Introduction

Endophthalmitis is an inflammatory reaction of intraocular tissue due to infection with pathogenic microorganisms, which can be a complication after cataract surgery and can cause permanent blindness. ^1^ The most common microorganisms that cause endophthalmitis after cataract surgery are coagulase-negative staphylococci (33–77%), *Staphylococcus aureus* (10–21%), and gram-negative organisms like *Pseudomonas* sp. are extremely rare unless there's an outbreak. ^2^

To minimize bacterial contamination and the risk of endophthalmitis, intracameral antibiotics can be used to reduce the natural flora of the eye, eyelids, and eyelashes before surgery. ^3^ The increasing worldwide concern about antibiotic resistance underscores the importance of using prophylactic antibiotics carefully to provide effective protection against endophthalmitis. This helps safeguard both patients and society from the emergence of bacterial strains that are resistant to multiple drugs. ^4^

French data from 2008 found that aspiration of the anterior chamber fluid during surgery revealed that bacterial contamination occurs in 2–46% of cataract surgeries using the phacoemulsification technique. ^5^

Intracameral antibiotic injection has become a favorable method worldwide even though it is still controversial due to a lack of level 1 evidence. ^6^ There is no standard prophylactic method of post-ocular surgery endophthalmitis. Intracameral antibiotic injection aims to achieve a high concentration of intraocular antibiotics in a short time to eliminate pathogenic microorganisms. ^7^

Endophthalmitis after cataract surgery is less common in countries where intracameral antibiotic injection is the standard method of prophylaxis. Before the use of intracameral cefuroxime, the incidence of endophthalmitis after cataract surgery was between 0.3 to 1.2%, and after its use incidents decreased to only 0.014 to 0.08 (January 2002 and December 2004, in Sweden) %. ^8^^,^^9^

The intracameral injection has several advantages; it easy to do, reduces the use of topical antibiotics, and addresses compliance issues with post-operative drug use by patients. ^10^^,^^11^ Fluoroquinolone has great potential as a prophylactic intracameral antibiotic injection as it has a wide spectrum, good penetration into the ocular tissue, and only mild side effects. ^7^^,^^9^^,^^12^

Levofloxacin is available commercially in Indonesia in the preparation of 5ml bottle of eye drops (Cravit ^®^, Santen Pharmaceutical, Japan). Levofloxacin 0.5% eye drops in 5 ml bottle has been clinically proven safe for intracameral injection in rabbits and human eyes undergoing cataract surgery. ^7^^,^^13^ Off-label intracameral levofloxacin 0.5% injection (Cravit ^®^) has been chosen as a standard prophylactic method in the Ophtalmology Department of Cipto Mangunkusumo Hospital. Levofloxacin 0.5% is also commercially available in a single dose 0.6 ml of sterile eye drops preservatives free (LFX ^®^, Cendo Pharmaceutical, Bandung, Indonesia), which is a local product in Indonesia. Currently, a commercially available single dose 0.6 ml of levofloxacin 0.5% eye drops has not been accepted as an intracameral antibiotic for humans because there was no safety study yet.

The objective of this study was to evaluate the safety of intracameral injection of levofloxacin 0.5% eye drop single dose 0.6 mL preservative-free (LFX ^®^) on rabbit eye. Rabbits were used as experimental animals in this study because certain parts of their eyes bear some similarities to those of humans and are easily observable. The rabbit’s eye globe and cornea are relatively large in size. Upon histopathological examination, the corneal epithelial layer comprises six layers of stratified squamous epithelium, with the deepest layer formed by cylindrical cells, similar to that of the human cornea. Similarly, the rabbit’s vitreous humor has a composition similar to that of humans, consisting of 99% water and 1% hyaluronic acid, resulting in a gel-like consistency.

## Methods

This study implemented an experimental design, where the animals were randomly allocated into three groups. The research was carried out at the Biomedical Research Center in the Health Research and Development Agency of the National Center General - Cipto Mangunkusumo Hospital animal laboratory, between April and June 2016.

### Ethical considerations

Ethical approval was obtained from the Ethics Committee of the Faculty of Medicine, University of Indonesia on 21 ^st^ March 2016 (No. 233/UN2.F1/ETIK). All efforts were made to ameliorate harm to the animals; all rabbits used as samples in this study underwent examination by a veterinarian and were declared healthy prior to the start of the research, followed by a 96-hour acclimatization process. The rabbits were individually housed during the study wire mesh cages measuring 50 × 40 × 35 cm, allowing them to move freely and minimizing stress. Food and water were provided ad libitum and the rabbits were monitored by experienced laboratory animal technicians. The cleanliness of the cages was monitored daily. Invasive procedures on the rabbits, specifically intracameral injections, were performed under semi-sterile conditions in the laboratory animal operating room, following aseptic principles to reduce contamination of the rabbits’ eyes. Rabbits that were euthanized at the end of the study were then incinerated. This article is reported in line with Animal Research: Reporting of in vivo Experiments (ARRIVE) guidelines. ^26^

### Animal model

To conduct a study on the effectiveness of levofloxacin in rabbit eyes, this study used 12 New Zealand White rabbits aged 3–4 months, with an average weight of 2.5–3.5 kg. The rabbits were obtained from Veteriner Laboratory Bogor, Indonesia. The rabbits were selected based on the inclusion criteria, which required no abnormalities in the cornea and anterior chamber and no signs of infection in the lids, anterior segment, and posterior segment of the eye before intra-camera injection. The research sample size was determined by the researcher to be 6 eyes per group, for a total of 24 eyes (12 rabbits), and treatment was given to 1 eye of each rabbit while the other eye served as a control. The selection of the sample size was based on the absence of supporting data from previous similar studies, as this research was a preliminary study, and thus the required values for sample size calculation were not available. Based on the consensus, an adequate research sample size for preliminary studies using animal models is six samples per group, following the ethical principles of the 3R (Replacement, Reduction, and Refinement) in animal research. Therefore, the minimum required sample size for each treatment group was determined to be 6.

The 12 rabbits were divided into two groups and randomly assigned to receive levofloxacin in one eye while the other eye received a balanced salt solution as a control. The first group received intracameral injection of a single dose of 0.5% levofloxacin in sterile eye drops without preservatives, while the second group received intracameral injection of levofloxacin 0.5% eye drops in 5 ml commercially available eye drops. All rabbits were reared according to institutional guidelines and the Association for Research in Vision and Ophthalmology Statement for the Use of Animals in Ophthalmology and Vision Research. The randomization method used was simple random sampling. The weight of rabbits in each group falls within the same range. The first author was aware of group allocation.

The rabbits were given general anesthesia through intramuscular injections of the hind limb muscles of ketamine hydrochloride (50 mg/kg) and xylazine (5 mg/kg), as well as a topical anesthetic of 0.5% tetracaine hydrochloride to ensure safety during the intracameral injection procedure. 30 minutes before the injection, 1% tropicamide eye drops and 2.5% phenylephrine hydrochloride eye drops were also used to dilate the pupils of rabbit eyes. A single drop of each solution was applied to both eyes to evaluate the posterior segment. Rabbits with bleeding in the anterior chamber after intracameral injection were excluded from the study, while rabbits that died or became sick before the end of the study period were included in the drop-out criteria.

### Experimental design

In total, there were 3 distinct groups of eye drops used:

‐ First group of LFX: 6 rabbits, 6 eyes
‐ Second group of CRAV: 6 rabbits, 6 eyes
‐ Third group of BSS: 12 rabbits, 12 eyes (combination of the 2 LFX and CRAV groups)

The surgeon used a 1 ml tuberculin syringe with a 30-gauge needle to perform all intracameral injections at the superior limbus. Prior to injection, 0.1 ml of aqueous humor was removed using a 30-gauge needle. The first group (LFX) of six eyes received an undiluted intracameral injection of levofloxacin 0.5% eye drops single dose 0.6 ml preservatives free, while the second group (CRAV) received a 0.1 ml injection of levofloxacin 0.5% eye drops 5 ml bottle preservative-free in the anterior chamber of the rabbits’ eyes. The third group received a 0.1 ml injection of balanced salt solution (BSS) (Alcon, Novartis Company) in the anterior chamber of the rabbits’ eyes. Therapy was administered by trained cage attendants under the supervision of a veterinarian as the cage supervisor. The exclusion criteria were eyes with hyphema and/or vitreous hemorrhage, and the dropout criteria were death or illness during the examination.

### Clinical examination

The ophthalmic examination of rabbits was conducted by a masked ophthalmologist who utilized a slit lamp (Kowa handheld slit lamp SL17) and indirect ophthalmoscope (Welch Allyn indirect ophtalmoscope) on the 1 ^st^, 3 ^rd^, 5 ^th^, and 7 ^th^ day after injection, with the resulting clinical examination scores being graded according to the scale ( Table 1). ^14^ All rabbits received anesthesia in the form of an intramuscular injection in the back of the thigh (hind limb muscle) before examination. The anesthetics given were ketamine HCl (50 mg/kg) and xylazine (5 mg/kg) by the veterinarian.

**Table 1.** Clinical grading scale.

Score	Cornea	Anterior chamber		Vitreous opacity
		Cell	Protein *flare*	
0	Normal	None	None	None
1	Mild stromal opacity	5–10	Mild	*Faint.* Vascular and retinal nerve can be identified
2	Moderate stromal opacity	11–10	Moderate. Iris detail and vascular can be identified clearly	Mild. Vascular detail and retinal can be identified but not nerve fiber.
3	Totally opaque	21–50	Moderate *Flare*, iris detail and vascular hard to be evaluated	Moderate. Vascular and retinal detail hard to be evaluated
4	-	>50	Severe. Frozen fibrin	Severe. Posterior segment cannot be evaluated.

### Histopathology examination

On the 7 ^th^ day after clinical examination, rabbits were euthanized with intravenous sodium pentobarbital 50 mg/kg. The eyes were immediately enucleated and fixed in 10% buffered formaldehyde solution for 48 hours. The eyes were sectioned sagittally into two parts, and then tissues were dehydrated and embedded in a paraffin block. The sections of 5 μm thickness were cut and stained with hematoxylin-eosin. A histopathology examination was performed by a masked pathologist with a light microscope (Olympus Microscope BX51). Histopathology scores were graded according to scale ( Tables 2 and 3). ^14^

**Table 2.** Corneal Histopatological scores.

Layer	Lesion	Score			
		0	1	2	3
Epithel	• Nuclear vacuolization • Nuclear condensation • Cytoplasmic vacuol	None	0–25% epithel layer	25–50% epithel layer	>50% epithel layer
Stroma	• Collagen matrix vacuolization • Keratocyt necrosis • Abnormal nuclear keratocyt • Cytoplasmic vacuol • Eosinophylic keratocyt	None	0–25% stromal layer	25–50% stromal layer	>50% stromal layer
Endothel	• Endothelial cell loss • Endothelial cell vacuolization	None	0–25% endothelial layer	25–50% endothelial layer	>50% endothelial layer

**Table 3.** Histopathology scores of anterior chamber, ciliary body, vitreous, retina and choroid.

Tissue	Lesion	Score			
		0	1	2	3
Anterior chamber	Inflammation	Normal	Mild Fibrin exudate	Moderate Fibrin, exudate	Severe inflammation
Ciliary body	Inflammation	Normal	Mild inflammation	Moderate inflammation	Severe inflammation
Vitreous	Inflammation	Normal	Fibrin and exudate	Partially-filled infiltrate	Full-filled infiltrate
Retina	Inflammation	Normal	Cystoid, thickened retina, mild neutrophil infiltration, and photoreceptor can be recognized	Retinal layer was recognizable, moderate neutrophil infiltrate, and photoreceptor is cannot be recognized	Retinal layer cannot be recognized Severe neutrophil infiltrate
	Retinal atrophy	Normal	0–25% retinal tissue	25–50% retinal tissue	>50% retinal tissue
Choroid	Inflammation	Normal	Mild inflammation	Moderate inflammation	Severe inflammation

### Statistical analysis

SPSS 20.0 for Windows was utilized to analyze the data, and either the One-way ANOVA or Kruskal-Wallis test was applied based on normality distribution during statistical analysis. The significance level of p<0.05 was deemed statistically significant.

## Results

### Clinical examination

There are 12 rabbits included in this experiment, with an average age of 4 months. Six rabbits were assigned to the LFX group, six rabbits were assigned to the CRAV group, and the other twelve were assigned to the BSS group. The mean body weight of the LFX and CRAV group were 3.13 kg, and for the BSS group, the mean body weight was 2.96 kg. No rabbits were excluded in this study.

Clinical score data are presented with a median and a maximum-minimum value for data distribution Shapiro-Wilk analysis results ( Table 4). ^24^ In the LFX group, a median clinical score indicated a score of 0 from the 1 ^st^ until the 7 ^th^ day. The maximum score found in the LFX group on the 1 ^st^ and 3 ^rd^ day was 1. Clinical scores were obtained from their mild stromal opacities of the cornea, without any other signs in the anterior chamber and vitreous. In the CRAV group, the median clinical score was 1 found on the 1 ^st^ day, while the median score on the 3 ^rd^ until the 7 ^th^ day was 0. The maximum score found until the 3 ^rd^ day in CRAV group for their mild corneal opacities was 1. In the BSS group, median clinical scores were 0 from the 1 ^st^ until the 7 ^th^ day. The maximum score of 2 was found on the 1 ^st^ day because of their cells and mild flare in the anterior chamber. Statistic tests of clinical scores between the three groups showed no significant differences. The statistical test used was the non-parametric k-independent samples Kruskal-Wallis test, because the data distribution was not normal, and the number of groups were compared to more than 2 groups.

**Table 4.** Comparison of clinical scores between the three groups. LFX= levofloxacin 0.5% eye drop single dose 0.6 mL preservative-free (LFX ^®^); CRAV=off-label intracameral levofloxacin 0.5% injection (Cravit ^®^); BSS=balanced salt solution.

Day	Group			*p*
	LFX Median (range)	CRAV Median (range)	BSS Median (range)	
1	0 (0–1)	1 (0–1)	0 (0–2)	0.11
3	0 (0–1)	0 (0–1)	0 (0–1)	1.00
5	0 (0–0)	0 (0–0)	0 (0–1)	0.61
7	0 (0–0)	0 (0–0)	0 (0–0)	1.00

**Figure 1.**
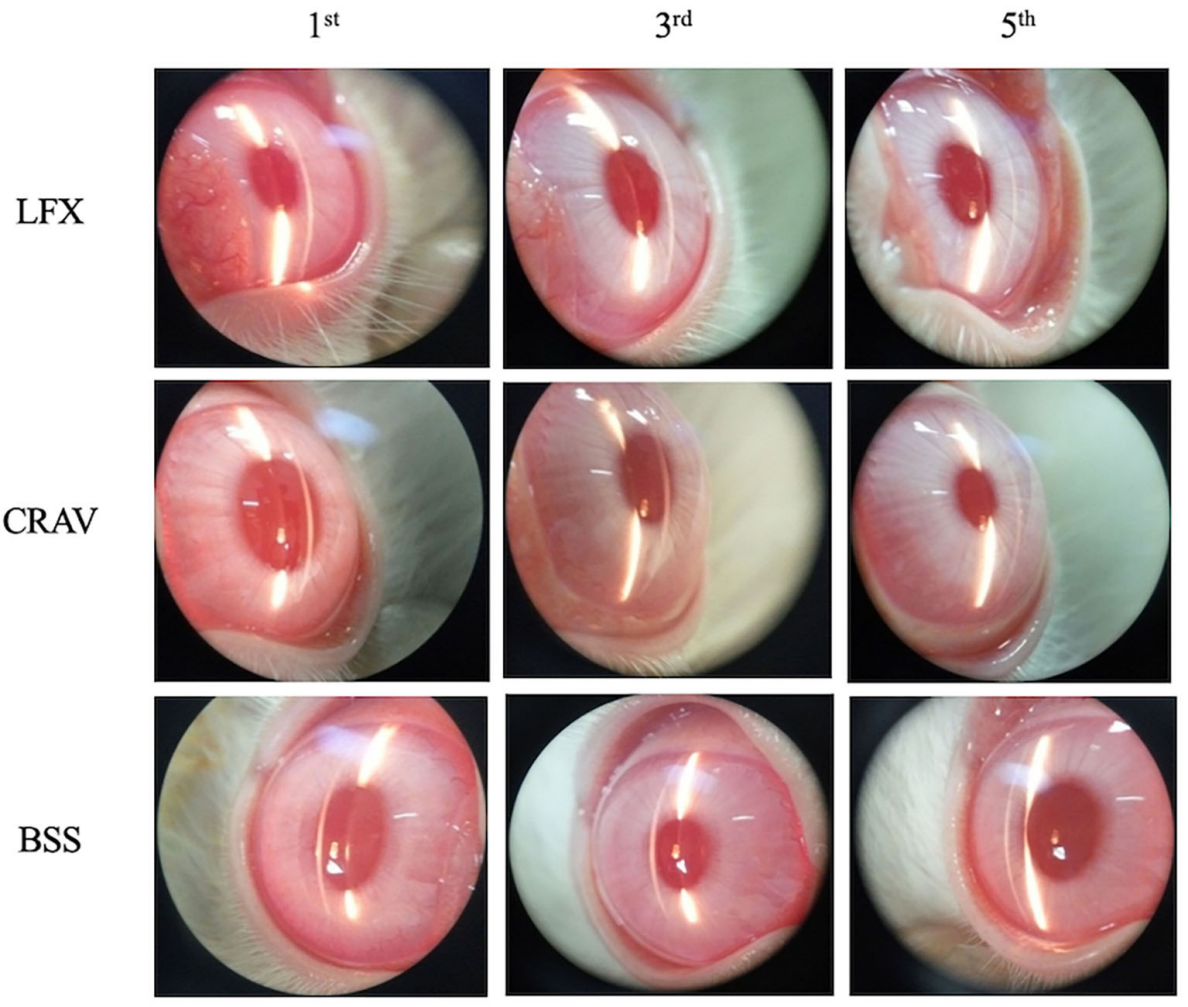
Clinical appearance.

The clinical presentation ( Figure 1) of the LFX group (left eye, rabbit No. 4) showed mild corneal opacities in the absence of cells and flare in the anterior chamber on the 1 ^st^ day. Corneal opacities disappeared on the 3 ^rd^ and 5 ^th^ day. Mild corneal opacities also appeared in the CRAV group (left eye, rabbit No. 7) and BSS group (right eye, rabbit No. 10) on the 1 ^st^ day. Corneal opacities were reduced on the 3 ^rd^ day and were cleared on the 5 ^th^ day, except the BSS group that still had very mild corneal opacities on the 5 ^th^ day.

### Histopathology examination

All three groups had the same median score of 3 with a range of 3–4 in the assessment of corneal histopathology. ^25^ Score 3 was obtained for their vacuolization of corneal endothelial cells that exceed 50% of the endothelial layer. Score 4 was obtained from corneal epithelial cell nuclei vacuolization between 0–25% (score = 1) and corneal endothelial cell vacuolization which exceeds 50% of the endothelial layer.

**Figure 2.**
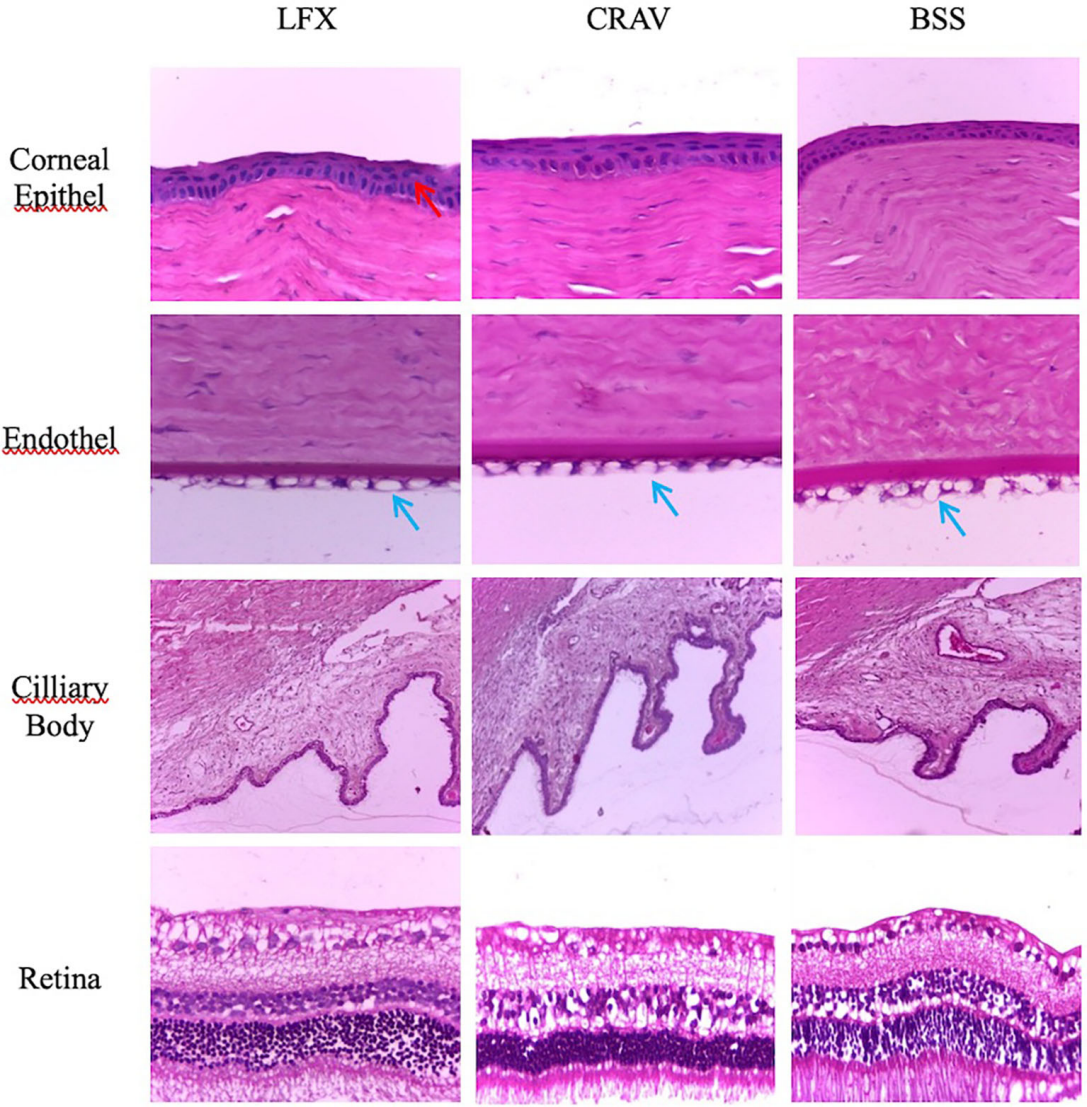
Histopathology appearance. No modifications (apart from arrows) have been made to these images.

Figure 2 shows a comparison of the histopathology picture of the epithelial layer of the cornea, corneal endothelium, ciliary body, and retina among the three treatment groups. Overview lining epithelium, stromal, endothelium of the cornea, and the retina were taken at 400× magnifications, while the ciliary body at 250× magnifications. Only mild epithelial cell nuclei vacuolization (<25% coating) was found in the cornea epithelial layer, as indicated by red arrows in group LFX. Corneal endothelial cells vacuolization (>50% layer) appear in all three groups (blue arrows). Vacuolization of endothelial cells is not accompanied by a loss of endothelial cells and did not look like a picture corneal stromal edema. The ciliary bodies in all groups were normal without signs of inflammatory cell infiltration. Retinas in the three groups were normal, with no signs of atrophy or inflammatory cell infiltration.

## Discussion

Off-label intracameral injection of antibiotics is very important to know the risk of toxicity. One way that can be done to find out the risk of toxicity is through non-clinical tests on experimental animals. ^15^ This study assessed the toxic effect from the structural and functional aspects of the levofloxacin intracameral injection. Functional aspects were assessed by the clinical score and structural aspects were assessed by the histopathology score. Comparison of clinical scores on the 1 ^st^, 3 ^rd^, 5 ^th^ and 7 ^th^ day post-injection showed no statistically significant differences between the three treatment groups. A study by Choi JA, *et al*. (2009) ^7^ also showed the result of clinical scores in the group of levofloxacin 0.5% did not differ statistically significant compared to the control group (balanced salt solution).

Maximum clinical changes post-injection were recorded in the form of mild corneal opacities or the presence of cells and flare light in the anterior chamber. Mild cells and flare were found in the eyes in the control group but not found in the two treatment groups. Kowalski RP, *et al*. (2015) ^16^ also reported mild clinical changes in the eyes of animal rabbits on the first day after intracameral injection of balanced salt solution. Clinical changes can also occur as a result of treatment and injured corneal. Corneal opacities can be caused by corneal edema. Corneal edema may occur as a result of the inflammatory process and/or a decrease in endothelial function. Inflammation contained in the cornea can be seen as inflammatory cell infiltrates (white lesions on the cornea). The decrease in endothelial function can be caused by the toxic effects of drugs or invasive treatments that damage the endothelial cells lining the cornea.

Invasive treatment in this study had been minimized by the use of 30G needles and injection locations near the superior limbus so as not to damage the central cornea. The toxic effects of antibiotics on corneal endothelial cells were known to occur until 2 hours post-exposure and the endothelial cell death rate was still high, although the drug concentration had been decreased. ^7^ Changes in corneal thickness can be measured quantitatively, but in this study it was not done.

A study by Kim SY, *et al*. (2018) ^17^ showed that central corneal thickness changes in the eyes of rabbits injected with 0.1 ml of levofloxacin 0.5% intracameral were not significantly different compared with the control group (BSS) since the first day post-injection. In the group of levofloxacin 0.5%, the average increase in corneal thickness was 9.00 ± 6.87 μm, whereas in the BSS group amounted to 14.83 ± 28.04 μm. Half of the volume aquos humor was eliminated in 46 minutes and finished in 2 hours. The concentration of levofloxacin 0.5% injected was 200 mg/ml in *aquos humor* after 2 hours. ^18^ The residual concentration, in theory, might be due to their mechanism of bonding with proteins in *aqueous humor*, and re-release process by surrounding tissues (cornea, iris and ciliary body). ^19^

Since the first post-injection day, all treatment groups’ vitreous and retinal appearances were normal. Abnormalities such as vascular changes, vitreous hemorrhage, retinal cotton wool spots, and pale area on the retina were not found. Uda *et al*. found that levofloxacin at a dose of 500 mg/ml was injected intravitreal directly did not cause signs of toxicity and no change of electroretinogram waves on rabbit retina was observed for 4 weeks. ^18^

Factors other than the concentrations that could affect the toxic effects of drugs are pH and osmolality. Corneal endothelial cells can be damaged if they are exposed to conditions that are outside of the pH range of 6.5 to 8.5 and the osmolality range of 200–400 mOsm/L. ^20^^,^^21^ BSS solution was formulated approaching physiological conditions with pH 7.5 and osmolality between 322–324 mOsm/L so it is safe to use intracameral without damaging the function of endothelial cells and does not induce an inflammatory response. Based on the products information, both preparations of levofloxacin 0.5% eye drops used in this study are both known to have a pH of 6.2 to 6.8 and osmolality 300 mOsm/L, but not with other deposits that are the secret formulation of the drug factory. ^16^ The incidence of a severe toxic anterior segment syndrome (TASS) has been reported after the use of intracameral moxifloxacin 0.5% eye drop. ^11^ Detergents and mucolytics are known to be the causes of severe TASS. This suggests there are other factors to consider in assessing the safety of intracameral injection ophthalmic antibiotic preparations.

The assessment methods of clinical scores in this study were semi-quantitative and relied on the ability of the examiner to observe clinical changes. Corneal opacities in clinical scores have not yet distinguished the detail and clinical changes due to toxic or inflammatory effects. ^7^ Another examination method is using specular microscopy to measure the corneal thickness, and to assess the density, variability, and hexagonality of corneal endothelial, which provides objective data of the structure and function of the cornea *in-vivo.* Unfortunately we did not have a specular microscope to carry out this examination.

Histopathology examination showed the presence of corneal endothelial cell cytoplasm vacuolization in more than 50% of the endothelial layer in all groups. Vacuoles appeared uniformly without any endothelial cell found missing. Vacuoles formed inside the cytoplasm are large enough to push the cell nucleus to the edge of the cell wall. Vacuolization of endothelial cells in this study could be said as not significant as a result of the toxic effects of drugs, and it also occurred in the control group. Vacuolization of corneal endothelial cells can be found after an 11 minute post-mortem on the cornea that is not fixed. The vacuolization continues to grow over time until the cell wall lysis occurs within 72 hours post-mortem. ^22^

Previous studies have used different methods to assess the toxic effects of drugs on endothelial cells to avoid factors such as post mortem changes. Choi *et al*. (2009) ^7^ used an electron microscope to find microstructural changes in the hexagonal shape of corneal endothelial cells exposed to the levofloxacin 0.5% after 7 days of intracameral injection.

Results of the histopathological assessment of ciliary body, retina, and choroid showed no sign of damage or inflammation caused by toxic effects in this study. Concentrations of levofloxacin ophthalmic preparations are still secure at a maximum of 3% (3000 mg/ml). Levofloxacin 10% (10,000 μg/ml) can damage tissue structures due to the toxic effect on the ciliary body in the form of edema and inflammation. ^18^^,^^23^ In Ref. 18, measured penetration levels of 0.5% levofloxacin ophthalmic preparations were injected intracameral. They found that after 2 hours, levofloxacin concentrations reached 30 mg/g in the anterior vitreous, 15 ug/g in the equator vitreous, and 10 μg/g in the posterior vitreous. Uda *et al*. (2014) ^18^ found that a dose of levofloxacin 500 mg/ml injected intravitreally did not cause retinal morphological changes and damage in experimental rabbits.

The limitations of this study are the subjectivity of clinical assessments, the limited aspects assessed, and the histopathology score being a modification score. To confirm the diagnosis of retinal disorders, it is essential to perform functional assessments such as visual evoked potentials and electroretinograms, as structural abnormalities of the retina do not always correlate with decreased visual function. Using fixation with a 10% formalin buffer solution does not prevent post-mortem changes in endothelial cells that rapidly occur as endothelial vacuolization. These differences need to be completed and confirmed by examination of endothelial cell function by specular microscopy examination.

In conclusion, the safety of 0.1 ml intracameral injection of levofloxacin 0.5% eye drops single dose 0.6 ml preservative-free is similar to 0.1 ml intracameral injection of levofloxacin 0.5% eye drops 5 ml bottle preservative-free in rabbit eye observed from the clinical score of the cornea, anterior chamber, and vitreous as well as the histopathological changes of the cornea, anterior chamber, ciliary body, vitreous, retina, and choroid.

## Data Availability

Figshare: Edwar, Lukman (2023): Main Table of Clinical and Histopatology Assessment Results.xlsx. figshare. Dataset. https://doi.org/10.6084/m9.figshare.22338343.v1. ^24^ This project contains the following underlying data
-
Table 1. Main Table of Clinical Assessment Results.xlsx -
Table 2. Main Table of Histopathological Assessment Results.xlsx Figshare: Histopathology appearance (Microscopic Images). https://doi.org/10.6084/m9.figshare.22640293.v2. ^25^ Figshare: ARRIVE checklist for ‘Safety of intracameral injection of levofloxacin 0.5% eye drops single dose 0.6 ml preservative free on rabbit eye’. https://doi.org/10.6084/m9.figshare.22638382.v1. ^26^ Data are available under the terms of the Creative Commons Attribution 4.0 International license (CC-BY 4.0).
