# Supplementary material for: Safety of intracameral injection of levofloxacin 0.5% eye drops single dose 0.6 ml preservative free on rabbit eye
Source: F1000Res. 2023 Dec 4;12:772. Originally published 2023 Jul 3. [Version 2] doi: 10.12688/f1000research.133293.2 (PMC10620477; doi:10.12688/f1000research.133293.2)
Supplement: Peer review history

# Reviewer report: Reviewer response for version 1

**DOI:** 10.5256/f1000research.146272.r184271

**Published:** 2023-11-01

**Authors:** Hasnah B. Eka

**Affiliations:** 1 Department of Ophthalmology, Faculty of Medicine, Hasanuddin University, Makassar, Indonesia

The abstract has explained well about this research carried out to the conclusion.

 The introduction has been well explained why this study must be carried out to find out whether this LFX drug can be used as cataract op prophylaxis to prevent endophthalmitis.

 The method chosen with the experimental rabbits was correct and the group formation with 2 controls was very good.

 The presentation of the research results is very clear by illustrating with photos and tables.

 Discussion of research results is very clear. It is only necessary to explain a little more about the endothelial vacuoles why they are also present in normal controls and whether these are also found in humans post-injection of antibiotics.

 Very good conclusion by directly answering the proposed title

Is the work clearly and accurately presented and does it cite the current literature?

Yes

If applicable, is the statistical analysis and its interpretation appropriate?

Yes

Are all the source data underlying the results available to ensure full reproducibility?

Yes

Is the study design appropriate and is the work technically sound?

Yes

Are the conclusions drawn adequately supported by the results?

Yes

Are sufficient details of methods and analysis provided to allow replication by others?

Yes

Reviewer Expertise:

Infection and immunology of eye, cornea, uveitis

I confirm that I have read this submission and believe that I have an appropriate level of expertise to confirm that it is of an acceptable scientific standard.

# Response

**Published:** 2023-11-20

**Authors:** Lukman Edwar

**Affiliations:** Ilmu Kesehatan Mata, Universitas Indonesia, Depok, West Java, Indonesia

Dear Hasnah B. Eka,

 Thank you for your insightful review. We appreciate your positive feedback on the clarity of our abstract, the well-explained introduction, and the appropriateness of the study design and methods.

 We appreciate your suggestion to provide more details on endothelial vacuoles in the discussion, especially regarding their presence in normal controls and potential relevance to humans post-antibiotic injection. 

 Thank you for your time and expertise in reviewing our work.

 Best regards,

 Lukman Edwar

# Reviewer report: Reviewer response for version 1

**DOI:** 10.5256/f1000research.146272.r184274

**Published:** 2023-07-19

**Authors:** Ovi Sofia

**Affiliations:** 1 Faculty of Medicine, Universitas Brawijaya, Malang, East Java, Indonesia

This study is supported by suitable and repeatable methodology, resulted in valuable findings that are important for clinical practice. However, there are some minor gaps that must be revised, that I mention by sections below.

**Introduction:**

 The clinical importance of intracameral antibiotics injection to prevent postoperative endophthalmitis should be highlighted in the first paragraph, in order to support the title and study objective.

**Methods:**

 It would be clearer if the positive findings showed in figure 1 and 2 are explained in sentences and indicated with arrow (if needed).

 There are some grammatical errors in vertical axis of figure 2. The sub chapter titles of statistical analysis and histopathology are considered inverted.

**Results:**

 Table 4 that has been put in Methods section was the comparison among study groups, so it should mentioned in Results section.

**Discussion:**

 No revision. The key findings of study are explained briefly and had been compared to the previous studies. The conclusion had mentioned at the end of the section and in line with the purpose.

Is the work clearly and accurately presented and does it cite the current literature?

Partly

If applicable, is the statistical analysis and its interpretation appropriate?

Yes

Are all the source data underlying the results available to ensure full reproducibility?

Yes

Is the study design appropriate and is the work technically sound?

Yes

Are the conclusions drawn adequately supported by the results?

Yes

Are sufficient details of methods and analysis provided to allow replication by others?

Yes

Reviewer Expertise:

Uveitis and intraocular inflammation, ocular infections, ocular surface disorders.

I confirm that I have read this submission and believe that I have an appropriate level of expertise to confirm that it is of an acceptable scientific standard.

# Response

**Published:** 2023-07-24

**Authors:** Lukman Edwar

**Affiliations:** Ilmu Kesehatan Mata, Universitas Indonesia, Depok, West Java, Indonesia

Thank you for being a reviewer for our article. We appreciate the suggestions provided, and we will gladly make the necessary improvements.
